# Crocodylian Head Width Allometry and Phylogenetic Prediction of Body Size in Extinct Crocodyliforms

**DOI:** 10.1093/iob/obz006

**Published:** 2019-03-23

**Authors:** Haley D O’Brien, Leigha M Lynch, Kent A Vliet, John Brueggen, Gregory M Erickson, Paul M Gignac

**Affiliations:** 1 Oklahoma State University Center for Health Sciences, 1111 West 17th Street, Tulsa, OK 74107, USA; 2 Washington University School of Medicine, 660 South Euclid Avenue, St. Louis, MO 63110, USA; 3 Department of Biology, University of Florida, Gainesville, FL 32611, USA; 4 St. Augustine Alligator Farm Zoological Park, 999 Anastasia Blvd, St. Augustine, FL 32080, USA; 5 Department of Biological Sciences, Florida State University, 600 West College Avenue, Tallahassee, FL 32306, USA

## Abstract

Body size and body-size shifts broadly impact life-history parameters of all animals, which has made accurate body-size estimates for extinct taxa an important component of understanding their paleobiology. Among extinct crocodylians and their precursors (e.g., suchians), several methods have been developed to predict body size from suites of hard-tissue proxies. Nevertheless, many have limited applications due to the disparity of some major suchian groups and biases in the fossil record. Here, we test the utility of head width (HW) as a broadly applicable body-size estimator in living and fossil suchians. We use a dataset of sexually mature male and female individuals (*n* = 76) from a comprehensive sample of extant suchian species encompassing nearly all known taxa (*n* = 22) to develop a Bayesian phylogenetic model for predicting three conventional metrics for size: body mass, snout–vent length, and total length. We then use the model to estimate size parameters for a select series of extinct suchians with known phylogenetic affinity (*Montsechosuchus*, *Diplocynodon*, and *Sarcosuchus*). We then compare our results to sizes reported in the literature to exemplify the utility of our approach for a broad array of fossil suchians. Our results show that HW is highly correlated with all other metrics (all *R*^2^≥0.85) and is commensurate with femoral dimensions for its reliably as a body-size predictor. We provide the R code in order to enable other researchers to employ the model in their own research.

## Synopsis

Alometria de Largura da Cabeça de Crocodilianos e Previsão Filogenética de Tamanho Corporal em Crocodilianos Extintos (Crocodylian Head Width Allometry and Phylogenetic Prediction of Body Size in Extinct Crocodyliforms)Tamanho corporal e mudanças em proporções corporais afetam amplamente os parâmetros da biologia de todos os animais, o que faz de estimativas precisas do tamanho corporal para táxons extintos um componente importante da compreensão de sua paleobiologia. Entre os crocodilianos extintos e seus precursores (por exemplo, os suchianos), vários métodos foram desenvolvidos para prever o tamanho corporal a partir de conjuntos de próteses de tecido rígido. No entanto, vários desses conjuntos têm aplicações limitadas devido à disparidade entre alguns grandes grupos de crocodiliformes e vieses no registro fóssil. Aqui, testamos a utilidade da largura da cabeça como um indicador de tamanho corporal aplicável a Crocodyliformes vivos e fósseis. Utilizamos um conjunto de dados de indivíduos machos e fêmeas sexualmente maduros (*n*=76), de uma amostra abrangente de espécies crocodílicas viventes, abrangendo quase todos os taxa conhecidos (*n*=22), para desenvolver um modelo filogenético bayesiano para predizer três morfometrias convencionais para tamanho: massa corporal, comprimento da abertura nasal e comprimento total. Em seguida, usamos o modelo para estimar parâmetros de tamanho para um conjunto específico de crocodiliformes extintos de afinidade filogenética conhecida (Montsechosuchus, Diplocynodon, e Sarcosuchus). Comparamos então nossos resultados com os tamanhos relatados na literatura para exemplificar a utilidade de nossa abordagem para uma ampla gama de fósseis asiáticos. Nossos resultados mostram que a largura da cabeça é altamente correlacionada com todas as outras métricas (*R*^2^≥0.85, para todas) e é comparável a dimensões femorais como um preditor confiável de tamanho corporal. Fornecemos o código R para permitir que outros pesquisadores utilizem o modelo em suas próprias pesquisas.Translated to Portuguese by J.P. Fontenelle (jp.fontenelle@mail.utoronto.ca)

Alometría del Ancho de la Cabeza de Cocodrilo y Predicción Filogenética del Tamaño Corporal en Cocodrilos Extintos (Crocodylian Head Width Allometry and Phylogenetic Prediction of Body Size in Extinct Crocodyliforms)El tamaño corporal y los cambios de tamaño corporal afectan ampliamente los parámetros de la historia de vida de todos los animales, lo que ha hecho que las estimaciones precisas del tamaño corporal de los taxones extintos sean un componente importante para comprender su paleobiología. Entre los crocodilianos extintos y sus precursores (por ejemplo, los suquios), se han desarrollado varios métodos para predecir el tamaño corporal a partir de conjuntos de indicadores de tejido duro. Sin embargo, muchos tienen aplicaciones limitadas debido a la disparidad de algunos grupos importantes de crocodiliformes y sesgos en el registro fósil. Aquí, probamos la utilidad del ancho de la cabeza como un estimador de tamaño corporal ampliamente aplicable en crocodiliformes vivos y fósiles. Utilizamos un conjunto de datos de individuos machos y hembras sexualmente maduros (*n*=76) de una muestra exhaustiva de especies existentes de cocodrilos que abarcan casi todos los taxones conocidos (*n*=22) para desarrollar un modelo filogenético bayesiano y predecir tres métricas convencionales para el tamaño: masa corporal, longitud del orificio de ventilación y longitud total. Luego usamos el modelo para estimar los parámetros de tamaño para una serie selecta de crocodiliformes extintos con afinidad filogenética conocida (Montsechosuchus, Diplocynodon, y Sarcosuchus). Luego comparamos nuestros resultados con los tamaños reportados en la literatura para demostrar la utilidad de nuestro enfoque en una gama amplia de tales fósiles. Nuestros resultados muestran que el ancho de la cabeza está altamente correlacionado con todas las otras métricas (todo *R*^2^≥0.85) y es conmensurable a las dimensiones femorales debido a su confiabilidad como predictor del tamaño corporal. Proporcionamos el código R para permitir que otros investigadores empleen el modelo en su propia investigación.Translated to Spanish by C.A. Alfonso (calfonsoc@vt.edu)

## Introduction

**Fig. 1:**
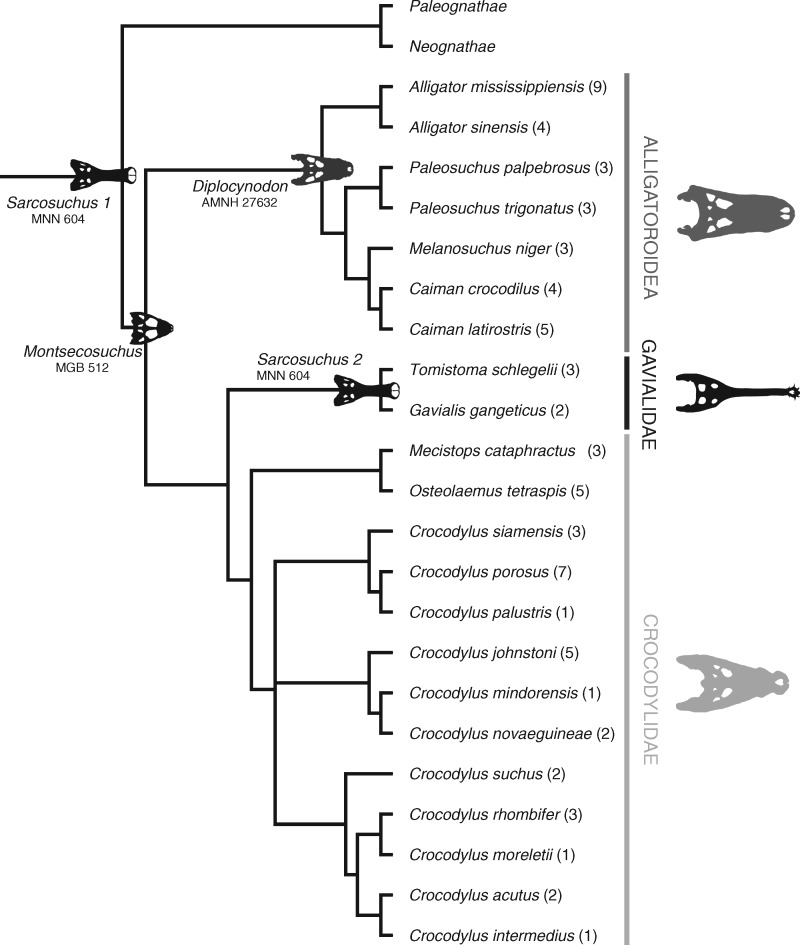
Likelihood-based molecular phylogeny utilized in the current study; modified from Erickson et al. (2012). Parenthetical values next to each taxon represent the number of individuals sampled; note that not all specimens have available TLs (see Supplementary Information S1). Fossil skull silhouettes are presented on the node to which they were grafted (with near-zero-length branch lengths). *Sarcosuchus imperator* was estimated from two phylogenetic positions: *Sarcosuchus 1* at the base of the phylogeny (conservative phylogenetic placement), and *Sarcosuchus 2* at the base of Gavialidae (longirostrine convergent placement).

For extant crocodylians (e.g., alligators, caimans, crocodiles, and gharials; Fig. 1), body size is an important determinant of ecology, evolution, and fitness (Seebacher et al. 1999; Grigg and Seebacher 2000; McNab 2002), as well as a primary factor influencing a range of life-history and functional/performance traits. Individuals that reach larger sizes during maturation gain higher bite forces and tooth pressures (Erickson et al. 2003, 2012, 2014; Gignac and Erickson 2015, 2016) and gain access to a wider variety of prey items (Abercrombie 1989; Abercrombie et al. 2001). Larger individuals generally show higher survivorship and reproductive rates, thus linking obtainment of larger within-population body sizes to increased fitness (Dodson 1975; Webb and Manolis 1989; Grenard 1991). Likewise, heterochronic deviations from ancestral body sizes (Gignac and O’Brien 2016; Godoy et al. 2018) can be largely linked to evolutionary shifts in craniodental disparity. Thus, body size in general—and body-size shifts, specifically—are significant for not only influencing modern crocodylian community dynamics and conservation, but can provide insights into the paleoecology of extinct Crocodyliformes (see Turner and Sertich 2010) and their archosaurian relatives, as well. For these reasons, understanding crocodylian allometry has been a perennial goal in vertebrate paleontology.

Numerous methods have been developed for the prediction of body-size parameters in crocodylians, such as body mass, snout–vent length (SVL), and total length (TL), from suites of hard-tissue proxies. These include vertebral column proxies (Seebacher et al. 1999; Young et al. 2016), pelvic dimensions (Prieto-Marquez et al. 2007), dental variables (Njau and Blumenschine 2006, 2011), head length (Webb and Messel 1978; Sereno et al. 2001; Young et al. 2011, 2016), depth of non-trigeminal cranial pits (Mook 1921; de Buffrénil 1982; de Buffrénil et al. 2015; Lynch et al. 2016), and length/circumference of the femur (Farlow et al. 2005; Young et al. 2011). Although these proxies generally show strong correlations with body-size metrics, there are scenarios and circumstances that may preclude their utility. For example, dental proxies may be less accurate when the tooth position is unknown (Drumheller and Brochu 2014); head-length is most useful within a single snout-shape ecomorph (Sereno et al. 2001 and references therein); and work on cranial pit-depths remains preliminary (e.g., Lynch et al. 2016). Femur length, the most commonly used metric, is difficult to reliably apply to studies of the fossil record, as extant crocodylians are semi-aquatic and many of their ancestors inhabited either fully terrestrial or fully marine habitats (Young et al. 2011). Differences in habitat and posture create differential mechanical loading regimes (Alexander 1989; Bonner 2006; Young et al. 2011), which can therefore render femur-size-based reconstructions tenuous between taxa, as well as across ecologies not represented among modern crocodylians. Similarly, the use of femoral circumference is best limited to intraspecific comparisons (e.g., developmental mass extrapolation; Erickson and Tumanova 2000). Finally, many of these proxies are restricted in paleontological contexts by taphonomy. Crocodyliforms commonly inhabited fluvial, costal, and shallow marine environments, which favor post-depositional compression (Reisdorf et al. 2012; Orr et al. 2016) and typically preclude three-dimensional (3D) morphological data collection of body-size proxies. To overcome these issues, we propose the use of head width (HW), measured as the linear distance between the lateral surfaces of the left and right jaw joints, as a viable alternative metric.

HW has been used as a body-size proxy on a case-by-case basis in a handful of studies examining extinct crocodyliforms (e.g., Kley et al. 2010; Gignac and O’Brien 2016). However, the scope of its utility has yet to be formally evaluated in a phylogenetically comprehensive context. For example, single-taxon studies of living crocodyliforms have identified HW, measured at the cranial platform (trans-quadratic width), as a primary contributor for discrimination of SVL (*Crocodylus porosus*, Webb and Messel 1978; *Caiman latirostris*, Verdade 2000) and a strong correlate with body mass (*Alligator mississippiensis*, Erickson et al. 2003, 2004; Gignac and Erickson 2015, 2016; Gignac and O’Brien 2016). Published data, therefore, suggest the utility of HW as a proxy for size. If a predictive relationship can be verified in an interspecific sample of living taxa, it would be advantageous for the study of fossil crocodyliforms, as cranial width is taphonomically resilient (Grigg and Kirshner 2015). The flat, durable bones and robust sutures of the crocodylian cranial vault are fortuitously resistant to diagenetic compression, whereas the elongate, round, and/or hollow structure of many postcranial bony elements renders them susceptible to crushing and deformation. Moreover, HW can be ascertained accurately using multiple techniques, rendering this value determinable even in fossilized specimens exhibiting fragmentary preservation (Gignac and O’Brien 2016). Although the utility of HW as a proxy for crocodyliform body size is demonstrable, its efficacy for prediction of body size metrics remains un-aggregated across broad taxonomic samples and unaddressed by phylogenetically-informed statistical methodologies.

Here, we measure the HW of sexually mature male and female individuals from a comprehensive sample of living crocodylian species and compare this value to three conventional metrics for “size”: body mass, SVL, and TL. Phylogenetic signal (Pagel’s lambda [Pagel 1999] and Blomberg’s *K* [Blomberg et al. 2003]) was found to be high for each metric, so we use phylogenetic generalized least squares (PGLS) regression to derive allometric equations for HW versus each size metric. These regressions were used to establish and evaluate the allometric relationship between HW and other body-size measures. To evaluate the predictive power of HW as a size proxy, we then iteratively estimate known values of mass for extant gavial individuals (Gavialidae: *Gavialis gangeticus* and *Tomistoma schlegelii*) in our dataset using the PGLS regressions and a Bayesian phylogenetic prediction framework. Finally, we use Bayesian phylogenetic prediction to estimate size parameters for a select series of extinct crocodyliforms with known phylogenetic affinity (*Diplocynodon hantoniensis*, *Montsechosuchus depereti*, and *Sarcosuchus imperator*). We compare our results to sizes reported in the literature to exemplify the utility of our size-estimation approach for a broad array of fossil crocodyliforms.

## Materials and methods

Institutional abbreviations: AMNH, American Museum of Natural History; MGB, Museo de Geología del Ayuntamiento de Barcelona; MNN, Musée National du Niger.

### Specimens

Data were collected for extant and extinct Crocodyliformes. Measurements of extant crocodyliforms were taken from 76 sexually mature male and female individuals representing 22 of the 24 currently recognized extant crocodylian species (Crocodile Specialist Group; King and Burke 1989; Erickson et al. 2012). Multiple individuals were available for 19 species; however, four species are represented by a single individual (*Crocodylus intermedius*, *Cr. mindorensis*, *Cr. moreletii*, and *Cr. palustris*). *Caiman yacare* was measured (four with HW values), but was not represented in the original phylogeny and is excluded from the phylogenetic analyses. All extant crocodylian data were collected from live individuals housed at the St. Augustine Alligator Farm Zoological Park, St. Augustine, FL, USA, and Crocodylus Park, Darwin, Australia (Florida State University IACUC Permit #0011 to G.M.E.). No animals were harmed during data collection. Extant specimen identification is available in Supplementary Information S1.

Extinct specimens were sourced from the literature and museum collections. We selected three specimens with accepted phylogenetic affiliation and with well-preserved crania. These include two stem neosuchians, *M.* (*Alligatorium*) *depereti* (Crocodylomorpha, Atoposauridae; MGB 512; Buscaloni and Sanz 1990) and *S. imperator* (Tethysuchia, Pholidosauridae; MNN 604; Sereno et al. 2001), as well as a crown taxon, *D. hantoniensis* (Alligatoroidea, Diplocynodontidae; AMNH 27632).

### Measurements

**Fig. 2:**
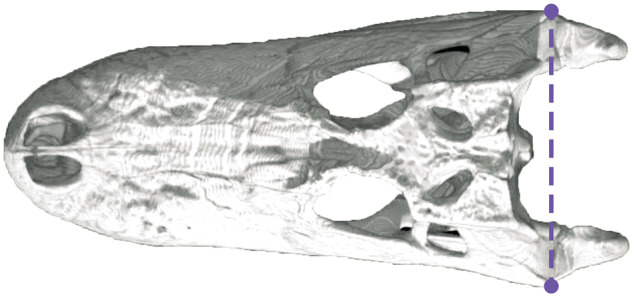
Skull of an American alligator, *Alligator mississippiensis*, in dorsal view, demonstrating the linear measurement for HW across the quadrates in purple (trans-quadratic width). For incomplete specimens, HW can be measured as twice the distance between the midsagittal plane and the lateral margin of the quadrate. Additional techniques are outlined in Gignac and O’Brien (2016). Skull accessed from Digimorph.org (Rowe et al. 2003).

Body size was represented by three metrics: body mass (kg), SVL (cm), and TL (cm). Body mass and SVL were reliably measured for all individuals; however, 20 individuals had incomplete tails and were excluded from the TL analyses, which resulted in the elimination of one taxon, *Cr. intermedius*. All individuals were captive males and non-gravid females. We elected to use a captive dataset due to large fluctuations in body weight among wild-caught individuals (Webb and Messel 1978). With a captive population, seasonal, dietary, and reproductive mass fluctuation are artificially controlled for. Mass of captive crocodylians is known to be approximately 25% greater than that of wild individuals of equal TL (Erickson et al. 2003, 2004). Therefore, all pertinent analyses were performed first with the raw measurements and again with a subsequent 25% mass correction to each individual’s measured mass. HW was measured directly as trans-quadrate distance to the nearest millimeter (after Gignac and O’Brien 2016; Fig. 2). Measurement data are available in Supplementary Information S1.

### Phylogeny

For our phylogenetic context, we used DNA sequence data collected by Gatesy et al. (2004), which was modified for tree-building by Erickson et al. (2012). Here, the tree is modified to rename *Crocodylus niloticus* to *Crocodylus suchus*, as the original specimens sampled have been re-identified as the latter. The original dataset consisted of published sequences for the nuclear genes RAG-1, BDNF, ATP7A, LDHa, c-myc, c-mos, DMP1, ODC, and 18S/28S rflp, and portions of the mitochondrial genes nd6, cytochrome b, the intervening glutamine tRNA, control region, 12S, and 16S (Gatesy et al. 2004; Erickson et al. 2012). These sequences were aligned using MacClade (Maddison and Maddison 2000) and treebuilding followed a maximum likelihood search using PAUP* (Swofford 2002) under a GTR+I+G model as indicated by Modeltest (Posada and Crandall 1998) and Akaike information criterion (AIC; Erickson et al. 2012). The resulting consensus tree was rendered ultrametric using penalized likelihood (Erickson et al. 2012). The phylogeny was further manipulated for this analysis to account for multiple measurements per taxon. Rather than averaging all measurements into taxon-representative values, we accounted for multiple individuals (sampling error) by representing each species as a hard polytomy of individuals (Christman et al. 1997; Felsenstein 2004; Housworth et al. 2004; Ives et al. 2007). Polytomies were constructed using Mesquite (version 3.10; Maddison and Maddison 2017) and were arbitrarily resolved by assignment of near-zero branch lengths when uploaded to the statistical program R (R Core Team 2016; package: ape [Paradis et al. 2004]). The addition of these near-zero branch lengths is necessary to accommodate analytical stipulations requiring a fully-bifurcating phylogeny while keeping added lengths too short to be registered as nucleotide or morphological differences. The phylogeny is available in Fig. 1 and Supplementary Information S2.

### Data inspection

Prior to data inspection, all variables were natural log-transformed to account for the nearly 500 kg range in mass among measured individuals. Because the analytical methods we employed are sensitive to a number of assumptions, we performed a series of data-structure inspection and regression diagnostics, using a suite of packages in R. First, we used Pagel’s λ (Pagel 1999) and Blomberg’s *K* (Blomberg et al. 2003) to calculate the phylogenetic signal of each measured variable (Table 1). Both metrics were calculated in {phytools} (Revell 2012). Regression diagnostics were implemented in the R packages {car} (Fox and Weisberg 2011) and {MASS} (Venables and Ripley 2002) by first fitting each value with HW in a linear model. Outliers were identified using the Bonferroni *P*-values for Studentized residuals (Cook and Weisberg 1982; Williams 1987). Both specimens of *G. gangeticus* were identified as outliers for TL and SVL, and the smaller individual of *G. gangeticus* was identified as an outlier for mass. We elected to retain these specimens in the analyses to maintain taxonomic representation, as well as to preserve representation of longirostrine (elongate- and extreme slender-snouted) forms (e.g., *G. gangeticus* and *T. schlegelii*), that are prevalent in the fossil record (Clark 1994; Brochu 2001). All regression diagnostics were replicated without *G. gangeticus*, and the results were not found to be significantly different (see Supplementary Information S3: R Code). Data were found to have sufficiently normal distributions using quantile–quantile plots of both fitted variables and residuals (Atkinson 1985; Fox 2008), and homoscedasticity (non-constant error variance) was found to be minimal (following Cook and Weisberg 1982). Data were therefore deemed appropriate for linear analysis without further transformation.

**Table 1:** Phylogenetic signal

	Total length		Snout–vent length		Mass		Head width	
Phylosig. statistic	Calculated statistic	*P*	Calculated statistic	*P*	Calculated statistic	*P*	Calculated statistic	*P*
Pagel’s lambda	0.917	***	0.939	***	0.92	***	0.879	***
Blomberg’s *K*	0.46	***	0.538	***	0.462	***	0.39	***

*** Indicates *P *≥* *0.0001.

### Phylogenetic generalized least squares

We analyzed the relationship between body size and HW using PGLS linear models (after Freckleton et al. 2002) performed using the R package {caper} (Orme et al. 2013). We also analyzed the relationship between mass and SVL as a benchmark for accuracy. We selected PGLS as our regression model due to phylogenetic non-independence in all variables. All variables were natural-log transformed prior to regression analysis. Because HW was treated as the dependent variable (*x*-axis), PGLS models were derived for each body-size metric as the independent variable (*y*-axis). Confidence and prediction intervals (CI and PI, respectively) for each model were calculated from phylogenetic variance–covariance matrices using the R package {evomap} (after Smaers [2014]; using the CI and PI estimation methods of Smaers and Rohlf 2016).

### Phylogenetic prediction of body size

We employed bivariate phylogenetic prediction following the BayesModelS methods of Nunn and Zhu (2014), implemented in R. This method uses a Bayesian algorithm to derive median trait value estimations and confidence intervals for an unknown variable in a specimen represented at a single phylogenetic tip (Garland and Ives 2000; Nunn 2011; Organ et al. 2011; Nunn and Zhu 2014). Both the measurable predictor variables and the degree of phylogenetic relatedness with neighboring taxa are used to predict trait values for unmeasured species. Previous studies have demonstrated that accounting for phylogeny increases the accuracy of trait prediction and better constrains 95% CIs and PIs (Garland et al. 1999; Garland and Ives 2000; Organ et al. 2007; Pagel and Meade 2007; Organ et al. 2011; Nunn and Zhu 2014). The underlying Bayesian framework results in posterior probability distributions that provide a mean estimation of each target trait, as well as a measure of predicted trait values that are higher or lower than the mean (Nunn and Zhu 2014). As developed by Nunn and Zhu (2014), BayesModelS draws phylogenies from a block of trees, such that phylogenetic uncertainty can be accounted for during trait estimation. For our phylogenetic predictions, we fixed *λ* and *K* to their trait-specific values from our estimations of phylogenetic signal.

#### Phylogenetic prediction of mass for extant Gavialidae

For preliminary verification of the accuracy of our allometric HW vs. mass regression model and Bayesian phylogenetic prediction methods, we iteratively predicted mass for five longirostrine specimens within Gavialidae (*G. gangeticus*, *n* = 2; *T. schlegelii*, *n* = 3). We used longirostrines for verification because they represent a unique ecomorph that led specimens of *G. gangeticus* to be identified as outliers. If Bayesian prediction is capable of accurately predicting mass from HW in gavials, then the algorithm is likely robust and thus suited for prediction of more common/conserved ecomorphs. For this analysis, we implemented an abbreviated draw, with 20,000 posterior values, a burn-in of 5000, and a thin of 100. Predicted and actual mass values were then compared using a Welch’s two-sample *t*-test. The predicted gavial masses have a mean of 130.05 kg, and the actual gavial masses have a mean of 129.75 kg (*P *=* *0.996). This indicates that the Nunn and Zhu (2014) method of Bayesian phylogenetic trait prediction is capable of accurately estimating unknown trait values even when predictions are made from taxa that are dependent variable outliers.

#### Phylogenetic prediction of size variables for extinct Crocodyliformes

Following extant verification, we used the linear models we derived from the extant-only sample to predict mass for three exemplar, extinct crocodyliforms of well-supported phylogenetic affiliation: *M. depereti*, *D. hantoniensis*, and *S. imperator* (see e.g., Turner and Sertich 2010; Bronzati et al. 2012; Pol et al. 2014; Wilberg 2015). Fossils were grafted onto the most conservative phylogenetic nodes possible (see Fig. 1) and given near-zero-length branches. As *D. hantoniensis* is directly related to extant taxa within Alligatoroidea, it was placed at the basal node of this clade. *Montsechosuchus depereti* is a stem eusuchian and was placed at the node ancestral to Alligatoroidea, Gavialidae, and Crocodylidae. As a pholidosaurid, *S. imperator* belongs to a neosuchian sister group of Eusuchia that cannot be included in our molecular/extant taxon phylogeny. We therefore grafted *S. imperator* to the base of the phylogeny. We elected to use the near-zero branch-length strategy to recognize the fact that it is not possible to determine the degree of shared molecular history between our fossil specimens and their extant relatives.

We selected these three specimens because they represent a large size-range, from one of the smallest crocodyliforms (*M. depereti*) to the largest (*S. imperator*) discovered to-date. We estimated mass values from both the uncorrected and 25% corrected regressions to provide liberal and conservative mass estimations, respectively. We calculated 2,000,000 posterior values, with a burn-in of 500,000 and a thin of 10,000. Outputs were evaluated using likelihood and 50% credible intervals. (see Nunn and Zhu [2014] as well as our Supplementary Information S3: R code for further details on the use and implementation of this analysis, including instructions for the prediction of crocodyliform size parameters using HW.)

In addition to fossil mass prediction, we calculated TL for all three fossil specimens. For *M. depereti* and *S. imperator*, TL estimation provides an additional avenue for verifying the accuracy of HW-based prediction, as these specimens have measureable TLs. The *M. depereti* specimen we examined (MGB-512) is a well-preserved skeleton, embedded in a limestone slab with minimal compression and distortion (Buscalioni and Sanz 1990). The axial column was interpreted by Buscalioni and Sanz (1990) to have a TL of 53 cm. We used phylogenetic prediction in attempt to derive the known *M. depereti* TL value from its HW. We also performed phylogenetic prediction for *S. imperator* in the context of convergent morphology, by placing its phylogenetic affinity at the base of the clade containing *G. gangeticus* and *T. schlegelii* (Gavialidae; Fig. 1). *Sarcosuchus imperator* is a longirostrine taxon; thus, although it is not phylogenetically affiliated with Gavialidae, the covariance between extant longirostrine size metrics and HW is likely a better representation of the covariance in body size and HW for extinct longirostrine forms, like *S. imperator*. In PGLS, phylogenetic relatedness is incorporated into the regression equation error term through a phylogenetic variance–covariance matrix. Therefore, we also estimated the mass and TL of *S. imperator* as a longirostrine taxon. Our reasoning was that the regression error term of *S. imperator* (its distance relative to the regression line) should be described as similar to that of other longirostrine ecomorphs. This non-phylogenetic placement is consistent with the approach of sampling modern longirostrine taxa to better inform the study of their ecomorphological counterparts from the fossil record (Sereno et al. 2001; Farlow et al. 2005). Finally, SVL is a commonly used size metric for living reptiles; however, it is not customary to calculate SVL for fossil taxa, due in part to the difficulty in estimating the location of the vent. We, therefore, elected not to produce estimations for SVL in our exemplar fossil taxa.

## Results

**Fig. 3:**
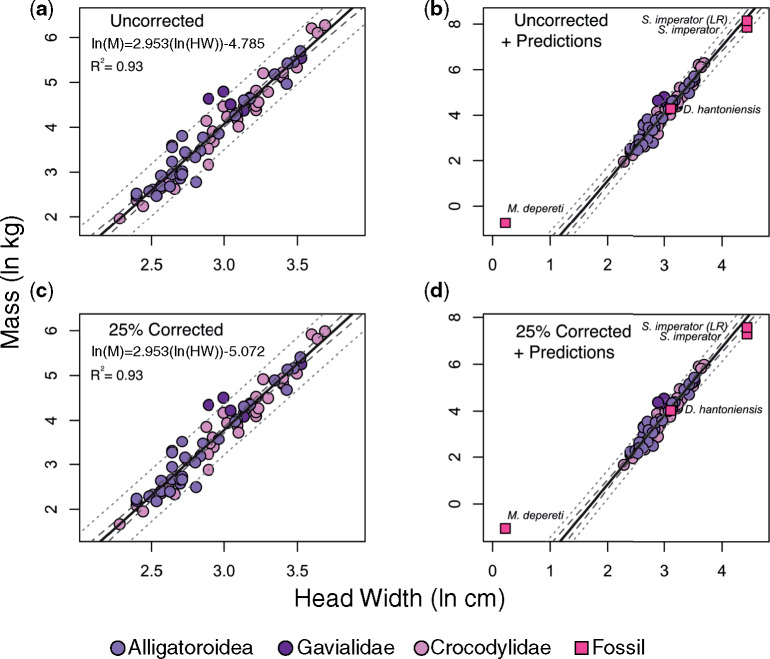
Regression plots quantifying the relationship between HW (cm) and mass (kg). In all plots, the regression line is solid, 95% confidence intervals are the longer dashed lines, and the 95% PIs are the smaller dashed lines. Note the different scales for each plot. Regression plot (**a**) demonstrates the relationship between HW and raw, uncorrected mass among extant taxa. In plot (**b**), fossil phylogenetic predictions derived from the regression equation in (a) have been added. Regression plot (**c**) demonstrates the relationship between HW and a 25% reduction in mass, to account for the mass discrepancy between extant captive and wild crocodylians. In plot (**d**), fossil predicted values derived from the regression equation in (c) have been added. *Sarcosuchus imperator* mass has been estimated from two phylogenetic placements: a conservative placement at the base of the phylogeny and a convergent ecomorphological placement at the base of Gavialidae, representing longirostrine forms. Abbreviations: HW, head width; M, mass; LR, longirostrine.

**Fig. 4:**
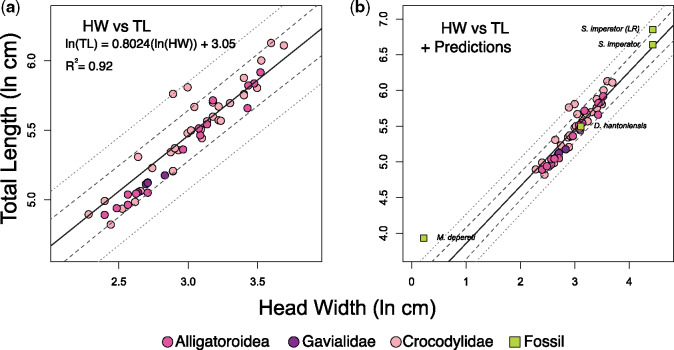
Regression plots quantifying the relationship between HW (cm) and TL (cm). In both plots, the regression line is solid, 95% confidence intervals are the longer dashed lines, and the 95% PIs are the smaller dashed lines. Note the different scales for both plots. Regression plot (**a**) represents the relationship between HW and TL among extant taxa. Plot (**b**) demonstrates the phylogenetic predictions of TL for extinct crocodyliforms, as estimated from the regression equation in (a). *Sarcosuchus imperator* has had TL estimated from two phylogenetic placements: a conservative placement at the base of the phylogeny and a convergent ecomorphological placement at the base of Gavialidae (with other longirostrine forms). Abbreviations: HW, head width; LR, “longirostrine”; TL, total length.

**Fig. 5:**
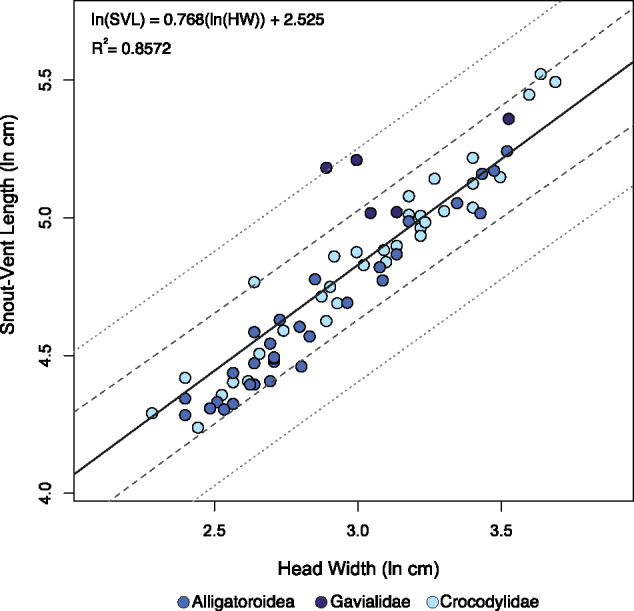
Regression plot quantifying the relationship between HW (cm) and SVL (cm). The regression line is solid, 95% confidence intervals are the longer dashed lines, and the 95% PIs are the smaller dashed lines. Abbreviations: HW, head width; SVL, snout–vent length.

The results for our anatomical measurements can be found in the Supplementary Information S1. PGLS regression statistics are reported in Table 2. We found that HW is highly correlated with the other metrics of body size (all *R*^2^ ≥0.85). Of the 76 individuals compared in our sample, three fell outside the 95% PI for HW compared with body mass. None fell outside of the 95% PI for HW compared with TL. One fell outside the 95% PI for HW compared with SVL (Figs. 3–5). Standard error was lowest for HW versus mass (0.251), indicating that observed values are closest to the fitted regression line and generate the narrowest 95% CI and PIs (Fig. 3). When slopes are considered, TL and SVL are negatively allometric with respect to HW (i.e., *b*_isometry_ =1; *b*_TL_ =0.802 ± 0.064, and *b*_SVL_ = 0.768 ± 0.075, respectively). Mass and HW have an isometric relationship (i.e., *b*_isometry_ = 3; *b*_M_ = 2.953 ± 0.193), whereas mass and SVL––the most common mass proxy––have a slightly positive allometric relationship (*b*_isometry_ =3; *b*_SVL_ =3.26).

**Table 2:** PGLS regression statistics

	HW vs. TL	HW vs. Mass*	HW vs. Mass^a^	HW vs. SVL	SVL vs. Mass*
SE	0.3104	0.251	0.251	0.283	0.473
df	53	69	69	69	69
Slope	0.80235	2.953	2.953	0.768	3.26
Intercept	3.05	−4.785	−4.785	2.525	−11.68
*P*-value	***	***	***	***	***
*R*^2^	0.9213	0.9311	0.9311	0.8572	0.9262
*R*^2^ adj.	0.9198	0.9301	0.9301	0.8551	0.9251
*F*-statistic	620.5	933	933	414.2	866.2

HW, head width; SVL, snout–vent length; TL, total length.

a Indicates mass with 25% reduction.

* Indicates uncorrected mass. Significance

*** indicates *P *≥* *0.0001.

We found phylogenetic signal to be significant across all metrics (*p_λ_* ≤1.0× 10^−7^ and *p_Κ_* =0.001 for all metrics; Table 1). PGLS results show that HW describes 93% of the variation in body mass, 86% of the variation in SVL, and 92% of the variation in TL (Table 2 and Figs. 3–5). The phylogenetic prediction results provide mean trait values, as well as upper and lower quartile estimations. All taxon-specific size prediction results can be found in Tables 3 (*M.**depereti*), 4 (*D. hantoniensis*), and 5 (*S. imperator*). The phylogenetic prediction estimated for *M. depereti*, the smallest crocodyliform taxon in our analysis, mass (uncorrected) ranges from 0.36 to 0.61 kg, with a mean of 0.47 kg. When the 25% mass reduction was applied to account for the fact that the fossil specimens are wild animals, these values were reduced to a range of 0.27–0.46 kg with a mean of 0.35 kg. The mean TL of *M. depereti* was estimated at 51.02 cm. For *D. hantoniensis*, the estimated uncorrected mass ranged from 61.45 to 84.78 kg, with a mean of 71.94 kg. Mass correction lowered these values to a range of 46.41–64.28 kg, with a mean of 54.39 kg. The TL of *D. hantoniensis* ranges from 234.07 to 254.33 cm, with a mean of 243.84 cm. When *S. imperator* size parameters are estimated from its conservative phylogenetic placement at the base of Crocodylia (position 1, Fig. 1), its size is roughly 25% smaller than when it is modeled as a “longirostrine taxon” (position 2, Fig. 1). Mean mass for *S. imperator* is predicted as a range between 1925 kg (base of the phylogeny and size corrected) to 3451 kg (convergent position at base of Gavialidae, without 25% mass reduction). The highest upper quartile reconstructed mass for *S. imperator* was 4296 kg (Gavialidae, mass uncorrected). When *S. imperator* TL is predicted from the conservative, non-convergent phylogenetic position, the mean estimate is 763 cm (approximately 25 ft); however, when in the convergent position (affiliated with Gavialidae), its mean TL is estimated 897 cm (approximately 29.5 ft). The highest upper quartile TL estimate for *S. imperator* is 947 cm (approximately 31 ft).

**Table 3:** *Montsecosuchus depereti* size estimations

	*Montsecosuchus depereti*		
	Lower quartile	Mean	Upper quartile
Mass^a^ (kg)	0.36	0.47	0.61
Mass^b^ (kg)	0.27	0.35	0.46
TL (cm)	47.79	51.02	54.55

a Indicates uncorrected mass.

b Indicates mass with 25% reduction.

## Discussion

Our phylogenetically-informed evaluation of HW as a body-size proxy comprehensively sampled extant adult crocodylians spanning a >75-fold range in body masses. This allowed us to test the accuracy of HW as a proxy for body size, compared with three standard metrics (mass, SVL, and TL). Our results show that HW meets or exceeds the precision of other linear measures, such as the most commonly used metric: SVL (Table 2). Because we sampled captive crocodylians exclusively, we incorporated a 25% mass correction (based on Erickson et al. 2003, 2004) directly into our regression models. Thus, our results include both conservative and liberal size predictions. The high *R*^2^ values returned in our PGLS results (0.86 ≤ *R*^2^ ≤ 0.93) justify the use of HW as a body-size proxy for Crocodyliformes, and one that is compatible with common biases in their fossil record.

The use of HW and phylogenetic prediction to reconstruct size proxies for exemplar fossil taxa, *M. depereti*, *D. hantoniensis*, and *S. imperator*, illustrates the applicability of HW-based models for evaluating and constraining estimates for a range of sizes and phylogenetic affinities. For our model, the mean estimation from the posterior draw represents the most probable value given the input predictor variable and phylogenetic relationship to crown Crocodylia. Because true values for mass are unknown, we attempted to validate the accuracy of the HW proxy using TL, which is a known variable when a complete skull and axial column are available. Buscalioni and Sanz (1990) previously measured the TL of *M. depereti* to be 53.0 cm for a “probably complete” (p. 250) adult axial column, rendering our mean estimate of 51.02 cm a reasonably accurate value (a difference of 3.7%).

We also estimated TL for a presumably adult *S. imperator*, which has a partially-complete axial column. Previous estimations for the size of this specimen have been calculated by multiple authors (Sereno et al. 2001; Farlow et al. 2005). Extrapolating from an estimated SVL of 571 cm (calculated following the linear equation for *Cr. porosus* log(SVL) of Webb and Messel 1978), Sereno et al. (2001) secondarily predict a TL of 11–12 m. Using minimum femur midshaft circumference, Farlow et al. (2005) predicted a TL of 724.6 cm for a specimen estimated to be 75% of a large *S. imperator* (MNN G102-2). This scales to 910.7 cm when additional growth is accounted for (Farlow et al. 2005). Compared with these available estimates, our conservative (base of Crocodylia) phylogenetic prediction for TL of *S. imperator* was under-predicted (mean TL =763.97 cm). When TL was estimated with *S. imperator* from a convergent phylogenetic placement (affiliated with Gavialidae; Position 2, Fig. 1), its 897 cm TL is less than 2% different from the scaled value of Farlow et al. (2005). Overall, our TL estimates are within 4% of known and previously estimated values for both *M. depereti* and *S. imperator*. Because the relationship between HW and TL has a similar *R*^2^ value (*R*^2^ =0.92; Table 2) as the relationship of HW and mass (*R*^2^ =0.93; Table 2), and therefore a comparable predictive power, phylogenetic prediction of mass from HW is expected to show a complementary degree of accuracy.

Because our mass reconstructions encompass conservative (25% mass reduction) and generous (uncorrected/raw captive mass) estimates, we can more directly compare our results with those of other taxa. The *D. hantoniensis* specimen has mass and TL estimates that are comparable to similar-sized *A. mississippiensis* individuals (Table 4 and Figs. 3 and 4). *Diplocynodon hantoniensis* is within the superfamily Alligatoroidea and has similar cranial dimensions and proportions as extant *A. mississippiensis*. Thus, this mass estimate is likely accurate. For *S. imperator* and *M. depereti*, taxa that are not members of crown Crocodylia, mass estimates must be compared with the 95% PIs, as well as estimations from the literature. With regard to CI and PIs, *S. imperator* lies within both the CI and PI and their upper bounds. Size for *M. depereti*, however, appears to be over-predicted (Fig. 3). While *M. depereti* does not have an estimated mass in the literature, *S. imperator* has had its mass estimated several times using different techniques (e.g., Sereno et al. 2001; Farlow et al. 2005).

**Table 4:** *Diplocynodon hantoniensis* size estimations

	*Diplocynodon hantoniensis*		
	Lower quartile	Mean	Upper quartile
Mass^a^ (kg)	61.45	71.94	84.78
Mass^b^ (kg)	46.41	54.39	64.28
TL (cm)	234.07	243.84	254.33

a Indicates uncorrected mass.

b Indicates mass with 25% reduction.

In the original description of the *S. imperator* specimen MNN 604, Sereno et al. (2001) estimated a body mass of 7960 kg based on extrapolating SVL to body mass relationships from extant crocodylians, *Cr. porosus* and *G. gangeticus*. In contrast, Farlow et al. (2005) estimated a mass of 2411 kg for a specimen (MNN G102-2) that is approximately 75% the length of the large adult *S. imperator* (MNN-604) using minimum femoral midshaft circumference. Based on the reporting by Farlow et al. (2005), this femur-circumference-based estimation would scale up to 3215 kg in a fully-grown individual. Our phylogenetically-conserved predictions of mass from HW are similar to that of the immature individual of Farlow et al. (2005), providing mean body-mass estimates of 1925 kg and 2589 kg (25% corrected and uncorrected, respectively; Table 5). Our mass estimates are, however, for a large, mature individual and therefore under-predicted relative to both Sereno et al. (2001) and Farlow et al. (2005). In an attempt to reconcile our estimate with this established literature, we also placed *S. imperator* as the last common ancestor of the only extant, fully-longirostrine clade (Gavialidae; Fig. 1). Our TL estimates suggest that such a placement may more accurately reconstruct size variables in *S. imperator* by exchanging phylogenetic accuracy for convergent ecomorphological reality. This scenario increases the highest mean mass estimate to 3451 kg. This value is approximately 200 kg above the scaled-up estimate of Farlow et al. (2005)––a difference of approximately 6%.

**Table 5:** *Sarcosuchus imperator* size estimations

	*Sarcosuchus imperator*		
	Lower quartile	Mean	Upper quartile
Mass^a^ (kg)	2045.19	2589.48	3330.98
Mass^b^ (kg)	1492.86	1925.04	2502.52
LR mass^a^ (kg)	2790.42	3451.45	4296.94
LR mass^a^ (kg)	1976.36	2416.77	2980.59
TL (cm)	715.54	763.97	813.8
LR TL (cm)	849.62	897.08	947.02

While broadly congruent with the femur-based estimates of Farlow et al. (2005), our mean and upper quartile mass estimates for *S. imperator* (Table 5) remain <60% of the 7960 kg value from Sereno et al. (2001). Even our 97.5 percentile estimates were unable to recover values near 7960 kg (see Supplementary Information S3: R-Script). This is a large discrepancy, which merits comparison of the methods presented herein with those presented by Sereno et al. (2001). Our predictions, and those of Farlow et al. (2005), are calculated directly from a single allometric predictor, whereas Sereno et al. (2001) estimated mass from a series of successive predictors. Sereno et al. (2001) first extrapolated SVL from linear equations derived from an intraspecific series of *Cr. porosus* (Webb and Messel 1978). They then used this point estimate to calculate mass from additional regression equations. This secondary step in their procedure is based on *Cr. porosus* specimens ≥41 cm SVL (group III of Appendix 2; Webb and Messel 1978), which have a higher allometric slope (3.2613) than specimens ≤20 cm SVL (group I of Appendix 2, slope of 3.0875) or from 21 to 40 cm SVL (group II of Appendix 2, slope of 2.0158) in the Webb and Messel (1978) dataset. If the three groups were combined to represent a more complete picture of *Cr. porosus* growth, the resulting overall slope would also be lower than the Group III value. Group III, therefore, harbors a steeper slope value (i.e., “adult bias;” Brown and Vavrek 2015) when compared with sampling a complete ontogenetic series. A straight-forward numerical example illustrates how even small decimal point deviations in slope can dramatically influence size estimations for extremely large individuals: The *Cr. porosus* growth series published by Erickson et al. (2014) has an SVL-to-body-mass scaling relationship of 3.1511. This slope decrease of just 0.11 would have resulted in a body-mass estimate of 3954 kg for *S. imperator* when otherwise following the Sereno et al. (2001) protocol. This mass estimate is 50% lower, and better aligned with the findings of Farlow et al. (2005) and those reported here. This underscores the need to be mindful of how decimal-level numerical differences can deceptively alter estimations when working with logarithmic power functions to predict extremely large values (Calder 1984). It also highlights the difficulty of estimating variables that are significantly outside the population represented in the source dataset.

At the smallest end of the size-spectrum, we estimate *M. depereti* to weigh between 0.27 and 0.46 kg (corrected mass estimates). These values lie above the 95% CI and PIs (Fig. 3), suggesting that HW may be less effective at predicting extremely small masses. Nevertheless, when compared with a developmental series of *A. mississippiensis* (Gignac and Erickson 2015, 2016; Gignac and O’Brien 2016) the estimated mass of *M. depereti* appears reasonable. Although the skull of the *M. depereti* specimen has closed sutures and it is thought to be an adult individual, its orbits are proportionally large for its skull length and width. Atoposaurs like *M. depereti* may therefore be pedomorphic (Tennant et al. 2016), suggesting that the use of an ontogenetic series may be best for atoposaur HW-to-mass predictions.

The use of Bayesian methods to generate posterior probabilities presents an advantage compared with other means of estimating body size in crocodyliforms, as this method returns a distribution of plausible values, confidence range, and a series of model-checking outputs (for details of the Bayesian prediction analysis, implementation, and interpretation, see Nunn and Zhu 2014). If there is sufficient biological evidence to presume that body size should be meaningfully greater or less than the mean estimate (e.g., terrestrial versus fully aquatic denizens, tail, or head size atypically large or small in a given taxon), the posterior draw is output in quartiles that provide an avenue to evaluate estimates higher and lower than the mean. For example, semi-aquatic crocodylians are habitat intermediates compared with their terrestrial (e.g., notosuchian) and fully aquatic (e.g., thalattosuchian) precursors. We, therefore, propose using outer quartile values for extinct (non-bipedal) taxa with clear habitat-related deviations from the body plans of living crocodylians. Specifically, lower quartile draws may be more appropriate for the masses of terrestrial forms, whereas higher quartile draws may be more appropriate for masses of marine forms (see discussions by Gearty and Payne 2018; Gearty et al. 2018). Taking this approach, future researchers would be able to account for phylogenetic position as well as habitat or body-type influences that are likely to alter HW–body mass relationships within the statistical framework utilized herein.

Body size is a critical parameter for addressing and comparing the biology of living and fossil taxa. HW, measured across the jaw joints, inherently encompasses the jaw adductor system and the brain—both of which are strongly conserved phenotypes among crocodyliforms (Iordansky 1964; Schumacher 1973; Sinclair and Alexander 1987; Busbey 1989; Cleuren et al. 1995; Endo et al. 2002; Holliday and Witmer 2007; Bona and Desojo 2011; Jirak and Janacek 2017). Likewise, the body plans and post-cranial anatomy of crocodyliforms are also conserved among all extant and most fossil groups (Grigg and Kirshner 2015). The stability of these features likely enables the predictive relationship between HW and other body size measures, even across large periods of evolutionary time. Moreover, HW returns values that are within 1–6% of estimates derived using femoral bone metrics––the current “gold standard” of crocodyliform body size proxies. Thus, the method outlined in this study for evaluating body size in fossil crocodyliforms reveals that HW is an appropriate proxy for numerous body-size measurements. We provide the R code as Supplementary Information S3 in order to enable other researchers to employ the model in their own research. Future work should further address techniques that target body plans not present among extant forms (e.g., terrestrial notosuchians, fully marine thalattosuchians) in order to hone the approaches proscribed in this study, including outer quartile draws, for comparisons across even broader samples of ecological diversity (e.g., Wilberg et al. 2019).

## Statement on human and animal rights

This research was carried out in compliance with Florida State University IACUC Permit No. 0011.

## Author contributions

H.D.O. designed the study, performed the analyses, wrote the paper, and developed the figures. P.M.G. designed the study, collected the data, performed the analyses, wrote the paper, and developed the figures. L.L. performed the analyses and wrote the paper. G.M.E. collected the data, and wrote the paper. K.A.V. and J.B. collected the data and revised the paper. The authors have no conflicting financial interests in the content or techniques discussed in this manuscript.

## Supplementary Material

Supplemental Information 1

Supplemental Information 2

Supplemental Information 3
