# Supplementary material for: Crocodylian Head Width Allometry and Phylogenetic Prediction of Body Size in Extinct Crocodyliforms
Source: Integr Org Biol. 2019 Mar 23;1(1):obz006. doi: 10.1093/iob/obz006 (PMC7671145; doi:10.1093/iob/obz006)
Supplement: Supplemental Information 2 [file obz006_supplemental_information_2.docx]

**Supplemental Information 2:** Molecular-derived phylogeny of extant crocodylians, in phylip format. Includes branch lengths and polytomies for species with repeated measures / multiple individuals. Available here: 10.6084/m9.figshare.7689170
