# Supplementary material for: Crocodylian Head Width Allometry and Phylogenetic Prediction of Body Size in Extinct Crocodyliforms
Source: Integr Org Biol. 2019 Mar 23;1(1):obz006. doi: 10.1093/iob/obz006 (PMC7671145; doi:10.1093/iob/obz006)
Supplement: Supplemental Information 3 [file obz006_supplemental_information_3.docx]

**Supplemental Information 3:** Folder containing executable R-script. Includes subfolder with all data files necessary to replicate the analysis presented herein, as well as the code for BayesModelS written by Nunn and Zhu (2014). Note that *Montsecosuchus deperetti* is coded as its synonym, *Alligatorium* (Vidal, 1915) in the included R-script. Available here: 10.6084/m9.figshare.7683719
